# Polyphenol Supplementation: Benefits for Exercise Performance or Oxidative Stress?

**DOI:** 10.1007/s40279-014-0151-4

**Published:** 2014-05-03

**Authors:** Kathryn H. Myburgh

**Affiliations:** Department of Physiological Sciences, Stellenbosch University, Private Bag X1, Matieland, Stellenbosch, 7602 South Africa

## Abstract

Supplement use among athletes is widespread, including non-traditional and biological compounds. Despite increasing research, a comprehensive and critical review on polyphenol supplementation and exercise is still lacking. This review is relevant for researchers directly involved in the topic, as well as those with a broad interest in athletic performance enhancement and sports nutrition. The purpose of this review is to present background information on groups of polyphenols and their derivatives because their differing chemical structures influence mechanisms of action; to discuss the potential of plant, fruit and vegetable-based biological supplements, high in polyphenol content, to affect exercise performance and biomarkers of oxidative stress and exercise-induced muscle damage; and to critically discuss the exercise studies and biomarkers used. Subjects in the studies reviewed were either sedentary, healthy individuals, or active, recreationally trained or well-trained athletes. Polyphenol supplementation in exercise studies included mainly extracts (multicomponent or purified), juices, infusions or an increased intake of polyphenol-rich foods. This review includes details of supplement doses and exercise test protocols. Many studies considered only the performance or one or two selected biomarkers of antioxidant capacity instead of a comprehensive choice of biomarkers to assess damage to lipids or proteins. Evidence is insufficient to make recommendations for or against the use of polyphenol supplementation (neither specific polyphenols nor specific doses) for either recreational, competitive or elite athletes. Polyphenols have multiple biological effects, and future exercise studies must be designed appropriately and specifically to determine physiological interactions between exercise and the selected supplement, rather than considering performance alone.

## Introduction

### Historical Context

Dietary supplements are used for a variety of reasons by athletes and recreational exercisers. Carbohydrate and protein intake may be increased to provide more energy or an anabolic stimulus. Some athletes may consider that their usual diet is insufficient in vitamins and minerals or that increased exercise requires a higher micronutrient intake than would be appropriate for a sedentary person.

Whether or not food intake was supplying sufficient vitamins and minerals was a serious topic of debate in 1942 at a meeting of the Royal Society of Medicine when the peace-time diet was compared with intake during the war when food rationing was in place [1]. The meeting reviewed evidence related to vitamin C, calcium and iron intakes. Of importance is that the concept of sufficient compared with optimal intake was discussed.

Skipping forward approximately 50 years to 1994, almost half of the athletes surveyed across 51 studies that encompassed a total of over 10,000 athletes used vitamin and mineral supplementation (prevalence 46 %) [2]. Some smaller studies reported that 100 % of subjects took supplements despite earlier research presenting little or no advantages [3]. More recently, surveys have included questions on the reasons for taking supplements. According to Petróczi et al. [4], the reported motives included “avoiding sickness, overcoming injuries and enhancement of diet”. Interestingly, these reasons bear a close similarity to the benefits observed when the war-time diet was supplemented with micronutrients.

### Current Context

A greater variety of supplements is now available to athletes. The two types of supplements with the highest use by Korean Olympic athletes were vitamins and oriental supplements [5] taken ‘to improve recovery ability’ (66 %) and ‘muscle performance’ (22 %). This change in the type of supplement and the reason for taking the supplement is not limited to athletes of oriental descent because athletes surveyed by a database in the UK also supplemented with *Echinacea* (prevalence of 31 %) and ginseng (prevalence 8 %) [4]. A study published in 2004 indicated that 17 % of collegiate female athletes in the US took non-traditional supplements [6]. This was similar to the use in adults older than 45 years in the general population (19 %) [7].

**Fig. 1:**
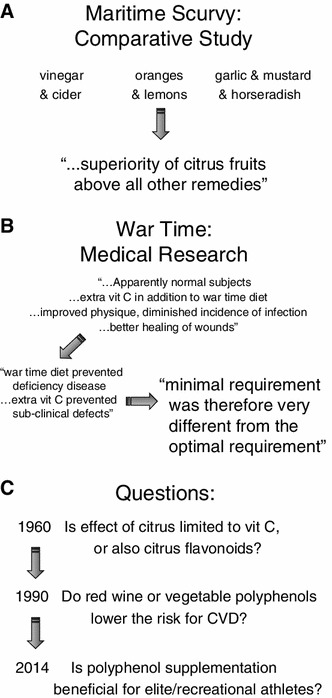
**A** Early studies noted the benefit of orange and lemon juice in preventing disease (scurvy) [8]. **B** Research carried out on civilians during war time indicated that the minimal requirement to prevent disease was different from the optimal requirement—supplementation resulted in better maintenance of physique despite food rationing [1]. **C** Even in 1960, water-soluble citrus polyphenols were more effective than vitamin C in preventing ‘injury’ in athletes [9]. The 1990s saw a surge in research attempting to define the constituents of the Mediterranean diet that conferred a benefit in reducing CVD. However, it has taken another 2 decades for scientists to gain a real interest in the potential effects of polyphenols on exercise performance.* CVD* cardiovascular disease

Supplements are now divided into traditional, less traditional and non-traditional supplements (see Table 1) [6]. Biological supplements typically contain chemical compounds extracted from fruit and vegetables, leaves, pods, roots and seeds. The idea that chemical compounds in fruit and vegetables, other than the vitamins and minerals, may have health benefits [1, 8] has been of long-standing interest (see Fig. 1) [9].

**Table 1:** Categories of dietary supplements [6]

Types	Categories	Examples
Traditional	Macronutrients	Carbohydrate, protein, fat
	Macronutrient components	Sugars, peptides, fatty acids
	Micronutrient	Vitamins and minerals
Less traditional	Functional foods	Foods with a particularly high content of a bioactive compound
Non-traditional phytochemicals	Oriental	Ginseng
	Herbal^a^	Parsley
	Botanical	*Echinacea*
	Biological^a^	Various polyphenols

^a^Substantial overlap

This review will focus on polyphenols and their potential benefit for athletes in the context of high-intensity endurance exercise, exercise requiring significant muscle power causing mechanical strain, or training stress. As polyphenol nomenclature is complex and frequently not used correctly, a relatively simple although comprehensive explanation follows.

## Polyphenols

### Classes, Derivatives and Sources

There are thousands of different plant polyphenols and hundreds of these are found in edible plants and plant products. The type and content of polyphenols differs substantially between different fruit, vegetables, leaves and seeds. Although most foods containing polyphenols have a variety of polyphenol constituents, they may be known best for the one with the highest proportion. Polyphenols are divided into family groups based on their chemical structure (reviewed by Bravo [10], and Manach and colleagues [11, 12]). There are 10 or more classes of polyphenols, but the four major classes are the phenolic acids, flavonoids, stilbenes and lignans [10]. Each of these has subclasses with hundreds of derivatives (see Table 2).

**Table 2:** Plant, fruit and vegetable-based biological compounds classified as polyphenols and separated according to differences in chemical structure [10–18]

Groups	Classes	Examples and derivatives	Excellent sources^a^
Phenolics	Cinnamic	Caffeic acid	Coffee beans
	Benzoic	Gallic acid	Tea leaves
Flavonoids^b^	Flavonols	Quercetin; kaempferol Quercetin glucosides^c^	Yellow onions, deep green vegetables, cherry tomatoes
	Flavones		Intensely flavored vegetables and herbs (celery, parsley)
	Isoflavones		Soy products
	Anthocyanins		Dark blue, red and deep red fruit and vegetables; red wine
	Flavanones^d^		Citrus fruit and pith of peel, concentrated tomato (paste)
	Flavanols	Naringin; hesperetin; eriodictyol; naringenin	
		Catechins and derivatives	Tannins, grape seeds, cocoa
		Epichatechin	
		Gallocatechin	
		Epigallocatechin	
		Epigallocatechin gallate	
		Proanthocyanidin	
Stilbenes	Resveratrol	Trans and cis-resveratrol	Wine, red grapes
	Piceid	Trans and cis-piceid	Red grape juice
Lignans		For example, enterolignans	Seeds, legumes, wholegrains, bulbs, certain fruit
		Allicin	

^a^Good sources: flavonols (broccoli, leeks, ‘oily pigment’ of citrus peel); anthocyanidins (aubergines, blackberries, black currants, black grapes); naringin flavanols (cocoa, green beans, apricots, cherries, green tea, grape seeds)

^b^Six of 13 classes

^c^Bioavailability greater than extract

^d^Naringin (grapefruit) bioavailability greater than hesperitin (oranges); eriodictyol (lemons)

The flavonoids are often referred to together, although there are at least six subclasses based on distinct differences in chemical structure [10]. Flavonols are present in a wide variety of fruits and vegetables. Quercetin and kaempferol are the most common flavonols in food [13]. They are present in a monomeric form and also as easily absorbed conjugates (e.g. quercetin glucosides); however, they are typically not found in abundant amounts per portion. Therefore, the intake is usually low, even with a healthy diet [14]. This may be a factor contributing to the popularity of supplements. Although present in wine and tea, flavonols are not the dominant polyphenol in these beverages. Rather, tea contains a high proportion of catechins belonging to the flavanol subclass, and wine contains more anthocyanins and proanthocyanidins.

Flavones and isoflavones are less common and are present in very specific foods (Table 2). Similarly, flavanones are present mainly in citrus fruit [14]. Classes of flavanones impart the characteristic tastes to the different citrus fruits; for example, naringin is found in high content in grapefruit and has a bitter taste. Citrus fruit is a good example of a food that cannot be considered as simply ‘high in flavanones’. Juicing significantly reduces the flavanone content in all citrus because higher levels are found in the white flesh below the skin and the separations between segments. The skin itself contains a relatively oily polyphenol, with the chemical composition placing it in the flavone subclass. Although it is a complex chemical analysis task to determine how many different polyphenols and derivatives are present in a juice (or other supplement), the level of one specific polyphenol can be determined more easily as only one standard would be required. Similarly, the bioavailability of one derivative of a polyphenol can be determined more easily than all the possible derivatives. Nonetheless, it is important to determine the overall bioavailability (total as opposed to only the unmetabolized form) because many different derivatives are formed in the gut before absorption.

Anthocyanins are flavonoids with several advantages but also one drawback. The first advantage is simply practical: fruit and vegetables containing high quantities of anthocyanins are easily recognizable as characteristically dark blue to black, red or deep red. The second advantage is that many foods containing anthocyanins have the highest amount of flavonoid per portion [11]. In fruits such as blueberries, the skin and pulp have a deep color and the skin is easy to eat. Black grapes and aubergines have an equally high anthocyanin content, but the skins must be consumed with the pulp. Unfortunately, it would seem that the bioavailability is relatively poor [12].

Flavanols are known best for the high content of catechins in chocolate and green tea. However, catechins and catechin derivatives are present in a wide variety of fruit and vegetables as monomers, modified monomers (e.g. epicatechin), or sometimes dimerized with other polyphenols such as gallic acid (gallocatechin, epigallocatechin, epigallocatechin gallate). Dimers, oligomers and polymers of catechins include the proanthocyanidins. Proanthocyanidins are found in a wide variety of fruit and vegetables, but are concentrated in the pods, beans and seeds of some fruit, vegetables and plants, and also in unripe fruit that has not yet ‘sweetened’ [10, 11]. Proanthocyanidins are often bitter, and impart the tannin taste to red wine and the bitter taste to cocoa [15]. In order to determine the uptake of the catechins in a supplement it is important to measure the conjugated catechins and the various metabolites as well, or the uptake will be severely underestimated [12]. For example, epigallocatechin gallate is found predominantly in the free form, whereas the other catechins may be methylated, sulfatated or glucuronidated. Therefore, all these forms must be quantified to get a true representation of the total. On the other hand, proanthocyanidins occur in many different polymer lengths, although these are acid labile, thus availability depends partly on the method of extraction [10].

Lignans are mainly found in oleaginous seeds and certain legumes, certain fruit and bulbs. The highest contents are found in linseeds, sesame seeds, lentils, cabbage, pears and garlic (allicin derivative) [16], as well as olive pulp and olive oil. Owen et al. [17] found that extra virgin olive oil has a substantially higher lignan content than refined olive oil, but that even among different extra virgin olive oils there is a lot of variability. Different types of grain contain widely varying quantities of lignans, and it is not yet clear why this is the case.

Stilbenes are uncommon in foods. Nonetheless, a well researched class is resveratrol and its derivatives, found in grapes and wine. Among red grapes and red wines, there are different levels of resveratrol derivatives, while red grape juice appears to have a higher content of the piceid derivatives than resveratrol [18]. Of interest is that the anthocyanin content of red wine is higher than the resveratrol content [19].

Polyphenol intake can be increased by conscious dietary choices of foods with high content, such as juice, hot drinks including tea infusions and coffee- and cocoa-containing beverages, or wine. As the content of polyphenols varies so much, supplements are an easier method to increase intake. Controlled intake does not exclude juice because juices can be made by reconstituting extracts and hence the exact content is known. Potential effects on performance and other issues of importance to athletes can be more easily determined by the ingestion of extracts. Even when known polyphenols are ingested in known quantities during laboratory tests, this does not account for the variation in bioavailability when ingested in different formats, or possible interactions with the concomitant intake of other nutrients. A further complication is that bioavailability will vary between individuals, thus necessitating the assessment in sufficient individuals for statistical confidence.

To assess the kinetics of uptake and elimination from the blood compartment, blood samples must be taken at regular intervals in order to calculate an area under the concentration-time curve [12]. The most important variables are the maximal concentration reached (*C*_max_), time to reach *C*_max_ in the blood and the half-life for elimination. An indirect assessment of tissue uptake could be urinary excretion, collected over a longer time period. Manach et al. [12] reviewed polyphenol bioavailability comprehensively, and critically assessed the data from studies of food, juice or supplement intake. There is a great deal of informative data presented from 97 studies covering 18 polyphenols, and it is beyond the scope of this paper to delve into the details. However, as a general observation it is clear that time to maximal concentration could vary from less than 1 h to over 6 h, which has clear implications for exercise studies. In terms of beverages, the reported duration to reach maximum uptake of flavanones from orange juice was relatively slow (~5 h), whereas flavanol uptake in tea and cocoa typically reached maximum between 1.5 and 2 h.

## Exercise and Antioxidants: Study Design Considerations

An interest in studying the effects of antioxidant supplementation on exercise performance and recovery is based on one or more of the following:

- Mitochondrial adenosine triphosphate production is not 100 % efficient, so that superoxide radicals are formed in increased quantities during exercise. The more oxygen is utilized during exercise the more superoxide radicals are formed that would need to be quenched.
- Muscle damage results in excess free radical production during the secondary phagocytic phase, and this prevents recovery.
- The endogenous mechanisms for removal of the excess radical species are insufficient and antioxidant supplements should prevent the negative consequences of excess accumulation.

### Exercise, Oxidative Stress and Antioxidant Supplementation

Reactive oxygen species are generated by several reactions during contraction, and play a normal and important role in muscle metabolism. This has been described by Reid in several reviews, including one in which both the positive and negative roles are well described in relation to exercise, force production and fatigue in human physiology [20]. Antioxidant supplements, including polyphenol-containing supplements, may benefit exercise performance directly or indirectly. Direct effects could involve the reduction of muscle fatigue at the level of contractile function. Reid [20] also examined certain dilemmas that have hindered a complete mechanistic understanding of basic research results that impact on the understanding of the potential direct effects of various antioxidant supplements. It is not within the scope of this paper to review the discussion, but it remains a seminal paper despite a decade of intensive research.

Indirect effects could include enhancement of training, reduction of physiological stressors that negatively impact on training (illness or the training response itself), or improvement in the ability to recover from training. Powers and Jackson [21] have discussed the evidence that at least low and physiologically normal levels of reactive oxygen species play roles in cell signaling and adaptation that would be expected to have positive outcomes. It is currently hypothesized that reducing the ‘cellular stress’ of exercise sessions may inhibit the endogenous adaptations to regular training [21].

Indirect influences on acute performance and adaptation in performance are very difficult to assess, particularly when the study period is long and includes the likelihood of a training effect in both placebo and supplemented groups. Therefore, to build a good reflection of the effect of a supplement on recreational and competitive athletes requires not only good study design to reduce confounding influences, but also a variety of studies with different designs to uncover different physiological and biochemical mechanisms.

### Protocol Considerations

Physiological markers (biomarkers indicative of physiological state or processes), although relatively easy to assay, have inherent biological variation within and between individuals. Biomarkers are frequently thought of in terms of broad groupings, including those for oxidative stress (see Table 3). However, they are usually derived from different reactions and hence also differ in time course of appearance and disappearance after exercise. Study design in terms of the timeline for blood sampling must take this into account as well as the pharmacokinetics of different supplements. Therefore, although a placebo-controlled, crossover study design is optimal and may sound like a simple and obvious choice, complex decisions must be made: pre-supplementation duration; timing of supplementation pre-testing; supplement dose, optimal washout period, etc.

**Table 3:** Terminology and biomarkers frequently used in exercise science research studies related to antioxidant properties [20–23]

Abbreviation	Definition	Description
Antioxidant power available in circulation^a^		
ORAC	Oxygen radical absorbance capacity	Comparison: radical scavenging ability of a standard antioxidant e.g. Trolox
TEAC	Trolox equivalent antioxidant capacity	Comparison: radical scavenging ability of a standard antioxidant e.g. Trolox
TAS	Total antioxidant status	Trolox, or other relevant antioxidant standard
FRAP	Ferric-reducing ability of plasma	
GAE	Gallic acid equivalents	Total polyphenol content using gallic acid as standard
CE	Catechin equivalents	Similar to previous
Evidence of lipid peroxidation or potential for lipid peroxidation		
LH	Lipid hydroperoxides	Early evidence of lipid peroxidation
MDA	Malondialdehyde	
TBARS	Thiobarbituric acid-reactive substances	Including MDA and other byproducts of lipid peroxidation
IsoP; iP	Isoprostanes	Resulting from free radical oxidation of arachidonic acid
Evidence of protein oxidation		
PC	Protein carbonyls	Determined using 2,4-dinitrophenylhydrazine assay
CO	Carbonyl groups	

^a^Could be used to assess the supplement itself (drink or dissolved powder extract)

In evaluating the literature, variables such as the type of exercise, exercise duration and intensity, and the type of subjects recruited are important considerations. For studies using elite athletes, it may also be important to consider whether an acute study was conducted in the off-season, early season or late in the competition season. The early season may be characterized by overload training, which places additional strain on antioxidant, immune and muscular systems [22, 23], systems that are of concern to athletes who report taking supplements. A training phase focusing on increasing muscle strength and power will change the ways in which muscles are recruited, and could result in the delayed onset of muscle soreness (DOMS). Plyometric training and eccentric weight training are both frequently used to improve muscle power and have also been used as a laboratory model to induce oxidative stress and exercise-induced muscle damage (EIMD).

EIMD usually causes DOMS, but they are not equivalent. They do not occur at the same time and DOMS does not accurately reflect the detailed physiological response to EIMD. DOMS will not be discussed as an outcome measure in this review. Primary EIMD results from excess mechanical forces experienced at the sarcomere level. These forces induce structural damage to the contractile and cytoskeletal proteins, and their dysfunction causes the loss of force associated with EIMD. Often the damage does not stretch across a whole muscle fiber, thus not always involving the membrane [24]. Secondary damage is complex but includes substantial damage induced by oxidative stress that is not restricted to the damaged area [25], although damaged membranes and proteins may be more susceptible to further free radical-induced damage. It is important to note that the parameters indicative of EIMD (reduced joint range of motion, force loss or elevated creatine kinase [CK] release) are not caused by oxidative stress. Therefore, reducing oxidative stress will not change the initial EIMD. Rather, the features of secondary damage make it more difficult to recover muscle performance capacity.

### The Precedent of Antioxidant Vitamins

Research using specific polyphenols as sports supplements has been preceded by many years of research for and against a beneficial effect of antioxidant vitamins (vitamin C, vitamin E, or the combination of vitamins C and E) on oxidative stress, performance and recovery [26]. This research has still not reached a point of consensus. As polyphenols also have antioxidant properties, it is useful to consider these controversies briefly, although further information on the topic should be sought from other reviews.

First, Knez and Peake [27] reported the results of a survey of the vitamin and mineral supplement intake of 37 ultra-endurance athletes. Of these athletes, 60 % used vitamin and mineral supplements, of which vitamin C intake was most common (97.5 %) and vitamin E intake was also high (78.3 %). Whether this vitamin intake in athletes is based on scientific consensus is questionable. Second, the potential of vitamins C or E to reduce acute oxidative stress or oxidative stress-induced damage to muscle or immune cells has been reviewed regularly since the early 1990s [26, 28–30]. The current controversy is whether vitamin C supplementation may negatively affect training responses such as improvements in oxidative energy metabolism and endogenous antioxidant mechanisms.

Endurance training is known to increase oxidative energy capacity and to decrease lactate production during exercise [31], and the mechanisms for mitochondrial biogenesis, although complex, are currently quite well understood at the molecular level [32]. Examples of contrasting studies include a study of amateur endurance athletes who participated in a placebo-controlled, 90-day supplementation study during their competitive season and ingested vitamin E (500 mg/day) and B-carotene (30 mg/day) daily plus vitamin C (1,000 mg/day for the final 15 days) or placebo [33], compared with a study on healthy sedentary men who participated in endurance training three times per week for 6 weeks while taking either 1 g vitamin C per day or placebo [34]. The athletes who adhered to competition season training programs all improved maximal oxygen consumption (*V* O_2max_) and peak cycle power output significantly. Despite similar maximal results between the placebo and supplemented groups, peak plasma lactate was lower and anaerobic threshold improved in the supplemented athletes, suggesting a beneficial effect of supplementation on submaximal oxidative metabolism [33]. The men who were sedentary at baseline had directly opposing results: no increase in *V* O_2max_ in the supplemented group but a significant increase in the placebo group [34]. Those and other studies have been reviewed by Nikolaidis et al. [35], and after thorough examination of the evidence (up to 2012), the authors concluded that unphysiological doses of antioxidant vitamin supplementation cannot be recommended, particularly on a daily basis for a long period of time. A study published in 2013 presented results showing that vitamin C and E supplementation within the physiological range (152 mg/day vitamin C and 50 mg/day vitamin E) did not reduce the training-induced adaptation in neutrophil endogenous antioxidant enzyme activities seen in the placebo group after 1 month [36]. It is clear that further dose–response studies are required and that it will be important to investigate effects in various physiological compartments.

Considering the above, this does not suggest that athletes can be given particularly convincing advice, and it is not surprising that there is still a quest to find new, potentially beneficial supplements outside of the traditional range.

## Polyphenol Supplementation and Exercise

### Polyphenol Supplementation and Endurance Performance

As the studies that will be discussed differ in study design, subject population, supplementation regimen and testing protocols, this review will pay attention to the details of these aspects and not only the outcomes of the various studies. The relatively recent advent of polyphenol supplementation for exercise studies has necessarily resulted in a fairly narrow range of doses. Nonetheless, a variety of polyphenol sources has been tested (see Table 4) [37–51]. On the one hand this is interesting, but on the other does not allow for much comparison as only quercetin testing has resulted in several studies.

**Table 4:** Polyphenol content of supplements taken before exercise trials

Polyphenol^a^	Reference	Dose	Pre-loading
Quercetin	[37]	1,000 mg/day	5 days
	[38]	500 mg/day	7 days
	[39]	1,000 mg/day	5 days
	[40]	1,000 mg/day	2 weeks
	[41]	600 mg/day	6 weeks
	[42]	1,000 mg/day	2 weeks
Juice/concentrate			
Pomegranate	[43]	260 mg/day	7 days
Chokeberry	[44]	35 mg/day	Training camp
Cherry	[45]	600 mg twice a day	3 days
Cherry	[46]	300 mg twice a day	7 days
Fruit/berry/vegetable	[47]	Not stated	1 month
Extracts			
Litchi fruit	[48]	200 mg/day	30 days
Grape	[49]	400 mg/day	30 days
Green tea	[50]	640 mg/day	1 month
Artichoke leaf	[51]	1,200 mg/day	5 weeks

^a^In studies including a supplement with multiple constituents, the main polyphenol has been presented here. More details are available in the text

#### Quercetin Supplements

Quercetin has a relatively long half-life in circulation, and most attention has been paid to this polyphenol in exercise-related studies. A recent meta-analysis provides a thorough summary of 11 different studies [52]. Of these, three peer-reviewed studies measured changes in *V* O_2max_, and although mean changes indicated improvements ranging from 2.3 to 7.5 %, the changes were not all statistically significantly different from placebo. The duration of supplementation did not separate the statistical differences between studies and, although the doses differed, this also did not seem to affect the outcome: after 5 days of supplementation with 1,000 mg/day, *V* O_2max_ increased by 7.5 % [37], and after a similar duration of supplementation (7 days) resulted in a lower percentage improvement of 3.9 %, which was statistically significant [38]. The main difference between the two studies was a possible dose–response effect because the second study using half the dose (500 mg/day) of the first also revealed approximately half the percentage increase in *V* O_2max_ of that of the first study mentioned. However, a third study with a similar duration of supplementation (5 days) at the higher dose of 1,000 mg/day reported no significant effect (+2.3 % with substantial deviation from the mean) [39], thus shedding doubt on the possibility of a dose–response effect. The subjects in those studies were untrained, active or moderately trained with an approximate mean *V* O_2max_ across the studies of 45 mL/kg per minute, but training status also could not explain the differing results.

It is important that two studies reviewed by Kressler et al. [52] were only available in abstract format (and thus are not cited here), but neither showed any improvement in *V* O_2max_. It should be considered problematical if non-significant results are not published (it is not possible to determine if the data contained in the abstract were never submitted or if they were not considered by referees to be of sufficient quality for one reason or another). For each of the unpublished studies, only nine subjects were used, but this was not really that different from three of the published studies (*n* = 11, 12 and 12 subjects). It is imperative that power analysis should be reported in studies with a small subject size, rather than omitting to submit such studies for peer review. Finally, for all peer-reviewed studies with even a subset of subjects previously reported in abstract format, this should be clearly indicated by the authors. This is particularly important in the digital age when abstracts are more readily available and may be used in a meta-analysis.

The above studies used the *V* O_2max_ test as their endurance performance test. One might question whether this is the appropriate endurance test for the assessment of polyphenols. Two other studies with possibly the most challenging and relevant endurance protocols reported significant effects on performance [40, 41]. Quercetin supplementation (1,000 mg/day) for 2 weeks improved performance by 2.9 % (*p* = 0.038) for a 12-min performance run at 15 % incline, undertaken directly after 1 h at 60 % *V* O_2max_ [40]. The volunteers were not trained and a distinguishing difference was that the supplementation period was 2 weeks, and the longer supplementation period may have been a factor contributing to the significance of the results. In the other challenging study with elite cyclists as volunteers, their 30-km cycling time trial (ergometer program included steep hill climbs) performance improved by 3.4 % overall, and the final 5 km of the same time trial indicated a 2 % improvement; thus, improvement was not limited to the initial half of the time trial [41]. In that study there was also a longer pre-loading period of 6 weeks before a crossover. The quercetin (300 mg twice a day) was administered in a drink that also contained green tea extract (300 mg twice a day) and a variety of vitamins (vitamin C 150 mg and vitamin E 50 mg twice a day). The effect of quercetin was not evaluated against a placebo, but against a control drink containing all the supplement constituents except for quercetin.

In summary, although the meta-analysis concluded that there were statistically significant although trivial effects of quercetin, the low-effect studies were with untrained subjects while the higher-effect studies were those with the most challenging protocols in well-trained subjects, thus still maintaining an open window for interpretation, but this would require further studies with larger subject numbers and dose–response effects included within the study design.

#### Fruit-Derived Supplements

Red wine contains the polyphenolic stilbene resveratrol and both have been studied extensively for health-promoting properties before exercise scientists became interested in polyphenols (see Fig. 1). In the field of exercise science, Bar-Or and Wilk [43] were the first to publish a study using grape juice as an intervention, albeit with interest focused on hydration in exercising children.

Polyphenol-rich juices employed as supplements in exercise studies include pomegranate juice [44], chokeberry juice [45] and cherry juice [46, 48]. As opposed to fruit juice itself, it has been more common to study fruit extracts high in polyphenols. For example, litchi fruit extracts (dimeric procyanidins and oligomeric proanthocyanidins enriched with catechins and epicatechins extracted from green tea: total 200 mg/day) were compared with vitamins C and E (800 mg/day plus 350 IU/day, respectively) and a placebo [49]. Healthy endurance-trained adults from the general population who were doing a minimum of 30 min of exercise three times per week were recruited. The mean *V* O_2max_ was above 60 mL/kg/min for each group. The study design was a three-group comparison without crossover, but there were 17–21 subjects per group. The litchi fruit extract had no effect on *V* O_2max_ after 30 days of supplementation. It is worth noting that vitamins C and E reduced *V* O_2max_ significantly, perhaps as a result of the non-physiological high doses, while placebo and litchi extract did not [49]. Therefore, all antioxidants cannot be considered to have the same metabolic or cellular adaptive effects.

Lafay et al. [53] supplemented with a grape extract (400 mg/day taken in capsule format). This extract did not contain one purified polyphenol. Rather, chemical analysis indicated that gallic acid, catechin and epicatechin were represented in the highest amounts. The double-blind, randomized, 1-month, crossover study was executed with a 2-week washout period. Despite good study design and subject numbers, the variation of sports in which they participated (handball, basketball, sprint and volleyball) resulted in large interindividual variations for most parameters tested. As 10 handball players represented a homogenous subgroup, these athletes’ data were analyzed separately and jump performance over 45 s was significantly improved with 30 days of grape extract supplementation.

In summary, there is insufficient evidence to conclude that polyphenol supplementation can improve endurance performance. However, there are also insufficient negative data to discourage its use actively for purposes other than endurance performance. Studies with submaximal testing and blood sampling for analysis of chemical parameters influenced by polyphenol supplementation will be discussed below (Sect. 5.1). Although, ultimately, athletes use ergogenic aids to improve performance (by definition), this does not imply that long-term influences on performance may stem from the influence of supplements on physiological processes that are more easily observed in the short term.

### Polyphenol Supplementation, Strength and Recovery

There is increasing interest in the effects of polyphenols in the context of muscle function and performance recovery after muscle micro-damage [44, 46, 47, 49]. This appears to be an area of research that is gaining momentum. For this type of exercise the rationale for using polyphenols is that:

- Exercise-induced excess radical production is too high for the endogenous scavenging mechanisms.
- Muscle micro-damage induces neutrophil oxidative burst.
- Breakdown of myoglobin results in production of ferric acid.
- Membrane lipids and proteins are damaged by oxidation reactions.

#### Fruit-Derived Supplements

In 2006, one of the first papers to assess effects of a fruit juice with high polyphenol content on recovery from eccentric exercise, was published by Connolly et al. [48]. In this study healthy untrained young men ingested tart cherry juice or placebo, twice per day for 4 days. Each bottle of tart cherry juice contained at least 600 mg of total polyphenols. On the fourth day, both groups performed two sets of 20 maximal eccentric biceps contractions using one arm. Supplementation continued for 4 days of recovery before a 2-week washout period and crossover. On the first day after the exercise intervention, average loss of strength with placebo was 30 % while the cherry juice ingestion sucessfully lessened the force loss to a decline of only 12 %. When supplemented, subjects experienced full recovery within 4 days, but this was not apparent with placebo. Pain was lower over the time course of the recovery period with the cherry juice supplementation. This early positive report has now been followed up by several other studies.

A second study using cherry juice supplementation, this time in well-trained subjects who participated in a variety of high intensity intermittent sports, compared the effect of the cherry juice concentrate with an isoenergetic synthetic fruit juice [46]. The supplement concentrate contained mainly anthocyanins and the amount ingested was approximately 300 mg twice a day for 7 days before the eccentric exercise test. The supplemented group improved the recovery of maximal voluntary knee extension force 24 and 48 h after the protocol of unilateral knee extensions (10 sets of 10 repetitions at 80 % of maximum voluntary contraction), which included a prolonged 3-s eccentric lowering phase [46]. The subjects regularly participated in resistance training as part of their sports conditioning. Subjects in a study reported by Trombold et al. [44], who were supplemented with pomegranate juice, were also accustomed to regular resistance training (although they were not athletes). Pomegranate juice was ingested twice a day for 7 days before and 7 days after the eccentric exercise intervention, which consisted of both three sets of 20 maximal eccentric elbow flexor extensions and six sets of 10 unilateral knee extensions. The total daily intake of anthocyanins was approximately 200 mg as well as 60 mg of ellagic acid derivatives. Isometric force loss of the elbow flexors was already highly significant at 2 h after the eccentric exercise test (35 % with placebo ingestion and significantly less—25 %—for the pomegranate juice). Force recovered somewhat in both groups 1 day later, with the difference between the pomegranate juice supplement and placebo still significant. DOMS was also significantly less 2 and 3 days after in the supplemented condition.

From these three studies alone, one might conclude that polyphenol-rich fruit juices are beneficial to reduce the extent of force loss, and may help recovery from high-intensity eccentric weight training bouts. However, this is not always shown as the subjects in the study by Trombold et al. [44] experienced no effect of the pomegranate juice supplementation on force loss or recovery from eccentric knee flexion. This could have been an influence of the size of the muscle group, but Goldfarb et al. [47] also reported no effect of a fruit/berry/vegetable juice concentrate on recovery after a protocol consisting of four sets of 12 maximal eccentric contractions of the biceps in which the initial loss of strength was 40 %. The latter study is more difficult to interpret for several reasons. First, the fruit and vegetable juice concentrates were dried to a powder and taken in capsules, and contained both polyphenols (flavonoids of unstated amounts, mainly anthocyanins) and antioxidant vitamins within the range recommended (7.5 mg of beta carotene, 276 mg of vitamin C, and 108 IU of vitamin E per day), and therefore the quantity appears to be appropriate. The loading period was 1 month rather than 1 week as in the other studies. The main factor in the protocol of that study was that the unloaded range of motion decreased substantially by 20–25° and no recovery was seen for the 3 days after the test; strength also did not recover, remaining as low as on the first day of ‘recovery’. The same research group had previously shown that the same supplement attenuated oxidative stress markers (oxidized protein and lipid marker biomarkers) after a 30-min run at 70 % of *V* O_2max_ in previously untrained women. This suggests that the supplement provided antioxidant protection and that it was the type and extent of the protocol that prevented an effect on EIMD.

In summary, in comparison to endurance performance trials, trials of recovery from muscle-damaging exercise protocols have been more promising. There appears to be sufficient evidence to continue this line of research in order to gain better insight.

## Polyphenols and Physiological Parameters

Polyphenols have antioxidant properties that can be measured directly in assays such as the oxygen radical absorbance capacity (ORAC), which determines the availability of water-soluble compounds with the ability to scavenge or quench oxygen radicals. Intense exercise decreases ORAC, whereas polyphenol intake increases ORAC; thus, it has been of interest whether polyphenol-based supplements can reduce the oxidative stress occurring during exercise relying on the oxidative energy systems. They also quench a variety of free radicals, thus not relying on a highly specific process like many of the endogenous antioxidant enzymes.

Physiological mechanisms underlying oxidative stress during exercise have been comprehensively reviewed by Powers and Jackson [21], while Peternelj and Coombes [54] highlighted controversies in the field. A short explanation of the categories of biomarkers currently in use, as well as typical nomenclature, are presented in Table 3. For the most part, the different biomarkers of ‘oxidative stress’ cannot be considered as interchangeable. For example, the capacity to quench free radicals is different from the products of oxidation reactions. Also, thiobarbituric acid-reactive substances (TBARS), although used as a biomarker of lipid oxidation, are non-specific and are also not very sensitive.

Some of the studies already mentioned above included submaximal testing and biomarker evaluation, while other studies had no performance test, reporting only the response of the biomarkers to exercise with polyphenol or placebo supplementation.

### After Submaximal Endurance Exercise

A carefully controlled, randomized, crossover study was undertaken in healthy untrained volunteers who ran for 1 h on a treadmill at 70 % *V* O_2max_ to test the effect of 200 g/day of purple sweet-potato leaf ingestion as part of the dietary intake [55]. Purple sweet-potato leaves have a high content of gallic acid and flavonoids (~33 mg/g and 427 μg/g dry weight, respectively). Pre-loading lasted 1 week and the washout period was 2 weeks. Blood samples were taken immediately before and after the exercise test and with follow-up 1 and 3 h after the exercise test. Supplementation resulted in a significantly higher blood polyphenol content that remained high immediately after exercise, but was reduced at 1 and 3 h after, no longer differing from the placebo trial. TBARS were highly significantly reduced with supplement pre-exercise and at 3 h after. These results are important because together they indicate that plasma polyphenol is involved in the resolution of oxidative stress, but that exercise still induced an element of oxidative stress that is important for the adaptation to exercise. Protein carbonyl (PC) concentrations were significantly different between the groups immediately post and 1 h after the exercise test, implying that proteins were protected from oxidative stress-induced damage at an earlier stage post-exercise. With these positive results in mind, the following paragraphs will compare other studies to determine how consistent these results may be in the literature.

Morillas-Ruiz et al. [56] supplemented elite cyclists with polyphenols in the form of a sports drink that contained a number of different potentially beneficial components: berry concentrates (total polyphenol consumed 2.3 g); vitamin C (20 mg/L); maltodextrin, pectin and whey protein (unspecified amounts), and vitamin B_1_ (15 % of recommended daily intake). The placebo contained vitamin C but no carbohydrate or other components in the supplement. Almost all the carbohydrate in the supplement drink was from the fruit concentrate (black grape [81 g/L], raspberry [93 g/L] and red currant [39 g/L]). Anthocyanin was the highest of the polyphenols present (759 mg/L), followed by hydroxycynnamic derivatives (246 mg/L) and ellagic acid (168 mg/L). After a standardized breakfast ingested 45 min before the trial, and the first drink 15 min before, the cyclists exercised at 70 % of *V* O_2max_; thus, a similar relative intensity to the study described above except that the test continued for 90 min as opposed to 1 h and took place on a cycle as opposed to a running test. The drink was consumed every 15 min, taking in 200 mL at a time. Similarities in results included elevated TBARS immediately after the exercise test (significant in the placebo, although only a tendency with the supplement) and carbonyl that was also only reduced with supplementation. As the supplement contained berry concentrates providing both polyphenols and carbohydrate, it is difficult to assign these differences to the polyphenols alone.

McAnulty et al. [42] compared the responses of three groups of athletes taking either a supplement containing quercetin and vitamin C as antioxidants (1,000 mg each), a placebo or a multicomponent supplement containing quercetin and vitamin C (1,000 mg each) plus additional flavonoids (400 mg isoquercetin, 30 mg epigallocatechin gallate) plus omega-3 fatty acids (400 mg). The effect of the supplements was determined from samples taken before supplementation and after 2 weeks of supplementation (pre-exercise). Thereafter, the athletes completed three exercise tests lasting 3 h each at 57 % of peak power output undertaken on 3 consecutive days, during which the supplementation was continued. Further blood samples were taken immediately post and 14 h after the third exercise test. Exercise decreased ORAC significantly, indicating that exercise induced an environment of oxidative stress. F(2) isoprostane increased significantly only in the placebo condition, indicating that arachidonic acid was broken down by cyclooxygenase, a reaction that was reduced for both of the supplement conditions. Also, lipid damage occurred only in the placebo supplemented group. The added flavonoids, vitamin E and omega-3 fatty acids did not add to the effects of quercetin plus vitamin C, probably because the antioxidant content was already so high.

### During Recovery from Eccentric Exercise

This section will discuss four studies in detail. The subjects varied from young and healthy men who participated in ‘recreational weight training’ [57] to healthy volunteers [39, 50] to athletes [46]. The studies will be discussed in order of the extent of repetitions included in the resistance exercise tests, starting with the study with the lowest volume.

The recreationally trained subjects performed four sets of bench press exercise (completing from 10 to 4 repetitions as the required percentage of one repetition maximum increased from 75 % to 90 %). This was done first after a week of water ingestion and again after a week with the ingestion of green tea infusion (10 mg of leaves per millilitre of water, steeped for 3 min; 200 mL, three times per day) [57]. This is not an appropriate study design because it was not a crossover study, and the first exercise test itself may have had an adaptive influence confounding the effect of the green tea. Tea can obviously not be ingested in a blinded fashion, but even a comparison of two groups of subjects taking either the placebo or the green tea would have allowed for some conclusions to be drawn. Nonetheless, the study does highlight important points. First, the interpretation of the biomarkers used: (1) Antioxidant capacity was assessed by assaying for the ferric-reducing ability of plasma, which was reduced significantly post-exercise after the placebo condition. A reduction in the ferric-reducing ability of plasma (or ORAC) after exercise indicates that the quenching ability is lower because some of the pre-exercise capacity has been utilized to neutralize the release of free radicals that occurred during the exercise. When there was no change in quenching capacity comparing pre- with post-exercise (such as occurred after the week of green tea supplementation), this indicates that none (little) of the quenching capacity had been used with the assumption that no (few) free radicals had been released. (2) Superoxide formation was assessed indirectly: the xanthine, hypoxanthine and urate metabolism pathway is activated by very high intensity exercise. This pathway results in superoxide formation as a byproduct. Green tea ingestion reduced the flux through this pathway at rest and the difference between the tea and water trials remained during the exercise test. (3) Similarly, pre-exercise lipid peroxidation was also reduced after the week of tea green supplementation and remained lower than control, 1 and 15 min after the bench press regimen. Important to note is the fact that lipid peroxidation was not actually affected by the exercise test under the placebo condition, hence these results can only be interpreted as changes to resting metabolism. The subjects were recreationally trained and the free-weight bench press comprised a considerable concentric contraction followed by a controlled eccentric contraction back to the starting position (unassisted). This was not sufficient oxidative stress to induce damage to lipids. From the study, it would appear that the potential health benefits of green tea were more apparent than a possible effect during exercise.

The exercise protocol in the study of Goldfarb et al. [47] also included four sets of eccentric elbow flexion, but intensity remained at 80 % with 12 repetitions for each set. Exercise-induced increases in both PC and malondialdehyde were prevented by the fruit/berry/vegetable polyphenol supplement, which had been ingested for 4 weeks. Even though malondialdehyde is a biomarker of lipid damage, lipid hydroperoxides were not increased in either group by the exercise regimen. Bowtell et al. [46] included more repetitions of single-leg knee extension at 80 % (10 sets × 10 repetitions), which is understandable because their subjects were athletes. Despite trained status, CK activity increased significantly as well as PC concentration in plasma 24 h after the exercise test. In particular, PC was significantly reduced with cherry juice concentrate ingestion [46]. In neither study did the polyphenol supplement reduce the exercise-induced increase in CK activity [46, 47].

The fourth study had a somewhat different objective: to assess the effect of green tea extract supplementation (640 mg/day polyphenols) for 1 month during a strength endurance training (a circuit of eight different exercises performed three times per session, 15 repetitions at 60 % one repetition maximum, three times a week) lasting the same duration, implemented in healthy subjects previously not trained [50]. The pre-training exercise test resulted in increased plasma hydroperoxides in the circulation. The 1-month training program, completely novel to the subjects, also increased resting lipid hydroperoxides in plasma, but only in the placebo group. One can conclude that training alone did not result in an endogenous mechanism to recover completely from the training days. However, the lipid damage biomarkers were neither elevated at rest nor post-exercise in the polyphenol-supplemented group. Also, significantly increased plasma CK activity 24 h post-exercise test was prevented only with the supplement. Therefore, training alone also did not prove sufficient to protect against EIMD.

That study seems to imply that more longer-term studies are required to obtain a better understanding of the potential benefits. Therefore, it is pertinent to review the few studies on elite athletes in which significant training forms part of the daily regimen of the research subjects.

### In Athlete Groups

During a 2-month period in the competition season, elite handball, basketball, sprint and volleyball athletes participated in a double-blind, placebo-controlled, crossover study, that is, 30 days on supplement and 30 days on placebo with a randomized order [53]. Slight, but statistically significant increases in biomarkers of lipid damage assessed in the urine (isoprostanes) occurred when athletes were on placebo, but not when on the grape extract that contained a variety of polyphenols (400 mg/day), at least half in the flavanol category. Resting CK activity levels in the circulation were high in these athletes, which is perhaps not surprising given the nature of their sports. There was a tendency (*p* = 0.1) for the supplement, taken for 1 month, to reduce CK activity. The mean value and variance between subjects were reduced with supplement (480 ± 81 U/L) compared with placebo (696 ± 177 U/L).

Oxidative stress biomarkers were assessed in a series of three studies with elite international rowers as subjects, with the research undertaken during training camps. The environment was thus very controlled and the training level very high. Assessing these studies in chronological order, the first used chokeberry juice (11.5 mg of anthocyanins in 150 mL of juice, three times per day) [45] as supplement, TBARS were significantly reduced 24 h following a 2,000 m rowing ergometer test. The second study used an artichoke leaf extract administered in capsule form (400 mg of extract three times daily) for 5 weeks [51]. Artichoke leaves contain polyphenols in the phenolic acid and flavonoid groups. Despite the higher intake of polyphenols compared with that in chokeberry juice, the study found that it had no effect on redox parameters assayed in red blood cells. The most recent study used a purified superoxide dismutase extract as supplement. Superoxide dismutase was extracted from melons and coupled with a biodegradable biopolymer, in order to maintain the antioxidant enzyme activity despite oral intake. It was administered twice daily for 6 weeks, also during a training camp [58]. The activity of this enzyme increased in red cells, but the major physiological influence was not on oxidative stress but on inflammation. C-reactive protein concentrations were highly significantly lower in the supplemented athletes at the end of the training camp.

The focus of this review was not on the inflammatory responses to the exercise test and whether or not this could be modified by polyphenol supplementation. Indeed, most of the studies discussed above did not include any inflammation biomarkers in their assessment panel. Another issue that is critical to remember is that examining results of parameters in plasma or serum may not reflect what is happening in immune cells or in tissues.

## Conclusion

The potential of polyphenols to have beneficial physiological effects is not a new concept. However, it has taken a long time for research in exercise science to gain momentum in this particular field. Although it is clear that polyphenol supplementation in a variety of forms and doses is able to increase the capacity to quench free radicals, at least in the circulation, it is not yet clear whether it holds beneficial effects for athletes. On balance of the current publications investigating the potential to have an effect on endurance performance, data in favor of polyphenol supplementation are sparse. However, protocols may not have been selected appropriately. Preliminary evidence suggests that it will be useful to continue the research on recovery from muscle micro-damage. This is a rich avenue for further research because plant and fruit-derived polyphenol compounds have complicated biological effects, and the interactions between these molecular effects and exercise remain to be elucidated.
